# A single cell survey of the microbial impacts on the mouse small intestinal epithelium

**DOI:** 10.1080/19490976.2022.2108281

**Published:** 2022-08-08

**Authors:** Derek K.L. Tsang, Ryan J. Wang, Oliver De Sa, Arshad Ayyaz, Elisabeth G. Foerster, Giuliano Bayer, Shawn Goyal, Daniel Trcka, Bibaswan Ghoshal, Jeffrey L. Wrana, Stephen E. Girardin, Dana J. Philpott

**Affiliations:** a Department of Immunology, University of Toronto, Toronto, Ontario, Canada; b Centre for Systems Biology, Lunenfeld-Tanenbaum Research Institute, Mount Sinai Hospital, Toronto, Ontario, Canada; c Department of Biological Sciences, University of Calgary, Calgary, Alberta, Canada; d Department of Laboratory Medicine and Pathobiology, University of Toronto, Toronto, Ontario, Canada; e Department of Molecular Genetics, University of Toronto, Toronto, Ontario, Canada

**Keywords:** Microbiota, scRNAseq, host–microbe interactions, germ-free, small intestine, intestinal epithelium

## Abstract

The small intestinal epithelial barrier inputs signals from the gut microbiota in order to balance physiological inflammation and tolerance, and to promote homeostasis. Understanding the dynamic relationship between microbes and intestinal epithelial cells has been a challenge given the cellular heterogeneity associated with the epithelium and the inherent difficulty of isolating and identifying individual cell types. Here, we used single-cell RNA sequencing of small intestinal epithelial cells from germ-free and specific pathogen-free mice to study microbe-epithelium crosstalk at the single-cell resolution. The presence of microbiota did not impact overall cellular composition of the epithelium, except for an increase in Paneth cell numbers. Contrary to expectations, pattern recognition receptors and their adaptors were not induced by the microbiota but showed concentrated expression in a small proportion of epithelial cell subsets. The presence of the microbiota induced the expression of host defense- and glycosylation-associated genes in distinct epithelial cell compartments. Moreover, the microbiota altered the metabolic gene expression profile of epithelial cells, consequently inducing mTOR signaling thereby suggesting microbe-derived metabolites directly activate and regulate mTOR signaling. Altogether, these findings present a resource of the homeostatic transcriptional and cellular impact of the microbiota on the small intestinal epithelium.

## Introduction

The intestinal epithelium represents a single layer of cells that forms a barrier between the inside and outside world.^1,2^ The collective community of trillions of diverse microbes is known as the gut microbiota, which maintains a mutualistic relationship with the intestinal epithelium. These host–microbe interactions have been well characterized to regulate host physiology and pathogenesis. Specifically, the relationship between the gut microbiota and the host intestinal epithelium has been well described to provide essential nutrients, process indigestible content,^3^ competitively inhibit opportunistic microbial infections,^4^ educate the immune system,^5^ and regulate host metabolism.^6,7^ With respect to pathogenesis, altered host–microbe interactions have been linked to the development of several pathologies, such as inflammatory bowel disease, cancer, diabetes, multiple sclerosis, and several other chronic inflammatory disorders. With such a large biological functional capacity, the gut microbiota has represented a potential therapeutic target in mediating and improving health.

Understanding how the epithelium facilitates this relationship has remained a challenge within the field due to the complexities in the architecture of the microbiota as well as the intestinal epithelium. The small intestinal epithelium is a rapidly regenerating tissue that is organized into repeating folds with villi extending into the lumen and crypts projecting toward the lamina propria.^8^ The single cell layer that covers the villi is composed of functionally diverse cells including enterocytes (ECs), goblet cells (GCs), tuft cells, and enteroendocrine cells (EECs).^8^ ECs account for most epithelial cells and facilitate the digestion and absorption of nutrients and water. GCs create a protective mucosal barrier through the secretion of mucins and tuft cells add to this protection by orchestrating a type 2 immune response against helminth infections.^9^ EECs secrete hormones to regulate intestinal growth, satiety, and metabolic processes.^10^ Intestinal stem cells (ISCs)^11,12^ reside within the crypts at the very base where they are intercalated by Paneth cells (PCs) that secrete antimicrobial peptides^13^ and provide growth factors and Wnt signals to ISCs.^14^ With distinct cellular localization and functions, defining the response of each epithelial population to microbes is vital in developing a mechanistic understanding of how the gut microbiota plays a role in health and disease.

In this study, we describe the transcriptional response of the small intestinal epithelium to microbiota by single-cell RNA sequencing (scRNAseq) of isolated epithelial cells from germ-free (GF) and specific pathogen-free (SPF) mice. We observed that the presence of microbiota increased the number of PCs within the intestinal crypt. Mapping the expression of pattern recognition receptors and their adaptors, highlighted the unique potential of different epithelial cell populations to respond directly to microbial ligands through *Tlr3, Nlrp6*, and *Nlrc4*. Differential gene expression analysis demonstrated that the microbiota strongly induces the expression of host-defense associated genes in several epithelial cell lineages, and the lack of microbes in GF mice induced genes involved in alternative metabolism pathways, suggesting a nutrient starvation stress response. Altogether, these findings help to paint a comprehensive picture of microbe-induced responses of the small intestinal epithelium at the single cell level.

## Methods

### Animals

GF and SPF C57BL/6NTac mice were maintained under respective standard GF or SPF conditions by the Division of Comparative Medicine (DCM) at the University of Toronto. GF mice were monitored for microbial contamination by aerobic and anaerobic bacterial culturing of fecal pellets in thioglycolate broth, brain heart infusion broth, and Sabouraud-dextrose broth for two weeks following euthanization and by assessing fecal DNA content by nanodrop following purification (NucleoSpin Soil, Macherey-Nagel). SPF mice were derived from GF C57BL/6NTac mice where female GF mice were orally gavaged with SPF mouse fecal content in 10% glycerol in PBS and mated to generate SPF mice from birth. F1 pups were mated, and the offspring were used as SPF mice for experiments. Sex and aged matched 8- to 12-week-old mice were used. Animal protocols were approved by the University of Toronto Committee on Use and Care of Animals.

### Small intestinal epithelial cell isolation

As previously described,^15^ whole small intestines from two GF or two SPF mice were pooled, flushed with cold PBS, and flared longitudinally. Small intestines were sectioned into 1 cm pieces and washed with cold PBS two times. Intestinal sections were incubated in 2 mM EDTA in PBS at 4°C for 40 mins with continuous shaking. Intestinal sections were washed with cold PBS and crypt enrichment was performed by vigorously shaking intestinal sections in cold PBS and passing the dissociated epithelial segments through a 70-μm cell strainer. Following centrifugation, enriched intestinal crypts were washed with cold PBS and incubated with 37°C trypsin (0.25% in HBSS) and mixed by gentle pipetting every 20 minutes for 50 minutes or until single cells were attained. The single-cell suspension was washed with ice-cold DMEM and passed through a 40-μm cell strainer. Trypan blue staining of the single-cell suspension was used to confirm >80% viability. An estimated 5000 cells were loaded for 10x Genomics single-cell isolation and library preparation following manufacturer’s recommendations. Illumina Hiseq 3000 was used to sequence the sample.

### Single cell RNAseq analysis

Sequences were processed with Cellranger v3.0.0 software (10x Genomics). Sequences were demultiplexed, unique molecular identifiers were aggregated and mapped to the mm10/GRCm38 transcriptome. Raw data generated by Cellranger were used to generate a SingleCellExperiment object. Scater^16^ was used to assess the quality of cells. Briefly, genes expressed in less than three cells were removed and cells with less than 1000 counts or 600 features, and more than 50% mitochondrial genes were removed. Quality control thresholds were chosen after assessing the impact of each threshold on all cell types. Gene expression data were normalized using Scran^17^ to compute cluster-specific size factors to remove cell-specific biases. The normalized gene expression data was merged into a Seurat object. In Seurat,^18^ the data were scaled, and principal components were determined using the top 2000 variable features. Datasets were integrated using Harmony.^19^ An elbow plot was used for the selection of principle components. Cells underwent unsupervised clustering using the shared-nearest-neighbor modularity optimization-based clustering algorithm (smart local moving algorithm). The optimal resolution for cell clusters was determined using the clustree package by increasing the clustering resolution by increments of 0.1 from 0 to 1. Cells were visualized using the uniform manifold approximation and projection (UMAP) method. Cell types were annotated using previously defined cell markers.^20^ Library size, unique features, and percentage of mitochondria-associated genes were used to remove low-quality cells (Supp Figure 1D, E). Thresholds for low-quality cell removal were selected by assessing the impact of each filter on broadly annotated cell clusters (Supp Figure 1B-E). Immune cells were filtered by expression of *Ptprc*. All scRNAseq analysis was performed using R 3.6.0.

**Figure 1.**
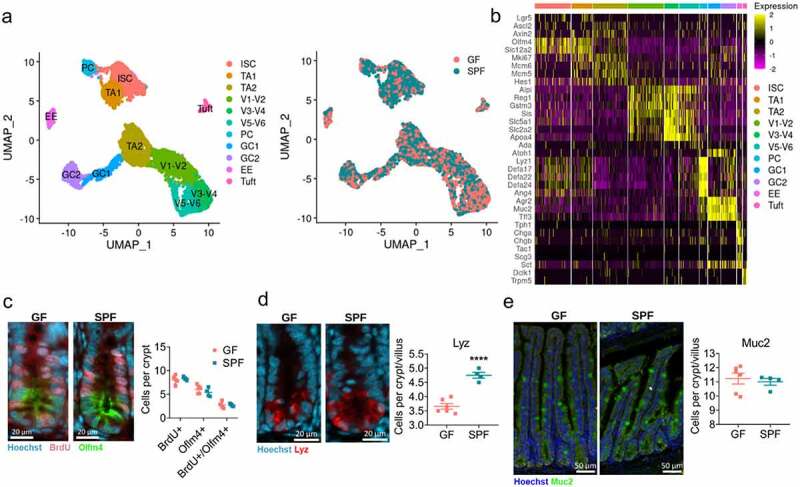
**Overview of the cellular landscape of the GF and SPF small intestinal epithelium**. A) scRNAseq cell clusters and distribution of integrated GF and SPF epithelial cells. B) Heatmap of marker genes used to annotate epithelial cell subsets C) BrdU and Oflm4, D) Lyz, E) Muc2 staining and quantification of jejunal/ileal small intestinal crypts from GF and SPF mice. Each point represents the average counts of 20 crypts or crypt-villus axis from one mouse (n = 4–6). Representative images shown. Bars represent mean ± SEM. ****, p < .0001 by two-tailed Student’s t-test.

### Trajectory inference analysis

scRNAseq data from GF and SPF mice were separated and an unsupervised trajectory inference analysis was performed using partition-based graph abstraction (PAGA)^21,22^ and RNA velocity.^23^ Principal component analysis was performed and an elbow plot was used to select the number of components to assess. Trajectory inference was rooted on the expression Lgr5 and Olfm4 for ISCs.

### Differential expression (DE) analysis

Differentially expressed genes were identified using the Seurat FindMarkers command for all genes. Ranked gene lists were generated by sorting on Log2FC. Genes not expressed in the GF and SPF datasets were filtered prior to gene set enrichment analysis (GSEA). GSEA was performed using ClusterProfiler^24^ against GO: Biological Processes (GO:BP). GO:BP gene sets assessed were a minimum of 15 and a maximum of 500 genes.

### Immunofluorescence staining & In situ hybridization

Distal 12 cm of small intestine were opened longitudinally, swiss-rolled, and fixed in 10% (v/v) formalin prior to paraffin embedding. Tissues were sectioned to 5 µm thickness, deparaffinized in xylene, and rehydrated with an ethanol gradient. Antigen retrieval was performed using 95°C sodium citrate buffer (pH6) for 30 minutes. Tissues were washed with PBS and blocked in 5% in BSA in PBS for 30 mins. Primary antibodies were diluted using 1% BSA in PBS and applied overnight. Primary antibodies were used at the following dilutions: Olfm4 1:1000 (39141 CST), Lyz 1:400 (EC 3.2.1.17 Dako), Muc2 1:500 (PA5-79702 Invitrogen), BrdU 1:500 (339810, BioLegend), Itln1 1:500 (MAB8074 R&D). Sections were washed three times in PBS prior to adding secondary antibodies. Secondary antibodies were diluted in 1% BSA in PBS for 30 minutes prior to Hoechst counter staining and mounting. Secondary antibodies were used at the following concentrations: anti-rabbit Alexa Fluor 647 1:1000 (A31573 Thermo Fischer), anti-rabbit Alexa Fluor 488 (A11008 Invitrogen), streptavidin eFluor 615 1:500 (42–4317-82 Thermo Fischer), streptavidin Alexa Fluor 488 1:500 (S11223 Invitrogen), anti-rat Alexa Fluor 647 1:500 (A21246 Life Tech), anti-rabbit Alexa Fluor 568 1:500 (A11036 Life Tech). In situ hybridization was performed using the RNAscope 2.5 HD Assay-Brown chromogenic assay with the following probe targets: *Nlrc4* (1031181), *Tlr3* (447791), *Nlrp6* (404561), *Ticam1* (806131). Tissues were counterstained with hematoxylin prior to ethanol/xylene gradient dehydration and mounting.

### 16S rRNA gene sequencing

DNA was extracted from fecal pellets using NucleoSpin Soil kit (Macherey-Nagel). The V4 hypervariable region of the 16S rRNA gene was amplified with a universal forward sequencing primer and uniquely barcoded reverse sequencing primer for multiplexing and sequenced at the Center for Analysis of Genome Evolution and Function (CAGEF) at the University of Toronto. The libraries were sequenced using the Illumina Miseq following manufacturer instructions. Fastq sequences were prepared using UPARSE.^25,26^ Paired reads were joined and trimmed. Qiime^27^ was used cluster sequences into operational taxonomic units.

## Results

### Single cell survey of the germ-free and specific pathogen-free small intestinal epithelium

To characterize the transcriptional response of the small intestinal epithelium to microbes, we performed scRNAseq on crypt-enriched whole small intestinal epithelium from GF mice and SPF mice. To normalize differences in genetics and establish physiological conventionalization from birth, a SPF mouse line was derived by oral gavage of GF female breeders with SPF fecal content. We annotated the fecal and ileal microbiota of our SPF mice used for scRNAseq by 16S rRNA sequencing (Supp Figure 1A). Crypt enrichment was performed to capture a greater proportion of ISCs and PCs, while simultaneously capturing all epithelial cell lineages along the crypt-villus axis.^15^ Following the removal of low quality and immune cells, we analyzed 2365 GF epithelial cells and 3272 SPF epithelial cells (Supp Figure 1D). This revealed 11 distinct clusters of epithelial cells that were labeled as ISCs, ISC-like transit amplifying cells (TA1), differentiating TA cells (TA2), PCs, two clusters of GCs (GC1, GC2), EECs (EE), tuft cells, and three clusters of mature ECs (V1-V2, V3-V4, V5-V6) based on established markers^20,28^ (Figure. 1A, B). Specifically, mature EC clusters were identified based on villus-axis zonation markers as identified by Moor et al. whereby *Reg1* marked villus bottom ECs (V1-V2), *Slc2a2* marked mid-villus ECs (V3-V4), and *Ada and Apoa4* marked villus tip ECs (V5-V6). Furthermore, while two TA cell clusters were identified based on their expression of cell cycle markers (*Mki67, Mcm6*, and *Mcm5*), TA1 had greater expression of ISC associated markers such as *Olfm4, Slc12a2*, and *Lgr5*, while TA2 had greater expression of enterocyte differentiation marker *Hes1* (Figure. 1B). Cells from either GF or SPF samples were well represented in each epithelial cell subset identified (Figure. 1A).

As previous literature suggests that microbes alter the cellular architecture of the small intestinal epithelium,^29,30^ we investigated changes in the abundance of ISCs (Olfm4+), TA cells (BrdU+), PCs (Lyz+), and GC (Muc2+). The presence of microbes did not impact the regenerative compartment of the crypts as no differences in abundances were observed in Olfm4+, BrdU+, or BrdU+Olfm4+ cells between GF and SPF mice (Figure. 1C). In line with previous observations, SPF mice had significantly increased numbers of PCs in the crypt compared to GF mice^29,30^ (Figure. 1D). No differences in abundance were observed in GCs between GF and SPF mice (Figure. 1E).

As PCs were increased in SPF mice, we performed trajectory inference analysis by PAGA and RNA velocity to investigate potential microbe-driven differences in epithelial cell lineage differentiation. PAGA and RNA velocity of all epithelial cell lineages showed the differentiation of ISCs to ECs or secretory lineages branched between the TA1-TA2 cluster and that this differentiation progression was the same between GF and SPF mice (Supp Figure 2A, B). In line with previous literature, ECs progressed from ISC to V5-V6 EC through TA cells and V1-V4 EC clusters.^28^ For better resolution of the secretory lineage progression, we performed trajectory inference on subclustered ISC, TA1, TA2, PC, GC1, GC2, EE, and tuft clusters. PAGA and RNA velocity showed similar secretory lineage progression between GF and SPF mice (Supp Figure 2C, D). Irrespective of microbes, PC are derived from ISC and TA1 clusters, while EE and GC clusters are derived from TA2, which arises from ISC and TA1 (Supp Figure 2C, D, E). Unexpectedly, GF tuft cells are more associated with GC2 lineage, while SPF tuft cells are more associated with ISCs (Supp Figure 2C, E). These data suggest that EC and secretory lineage differentiation progresses independently of microbes.

**Figure 2.**
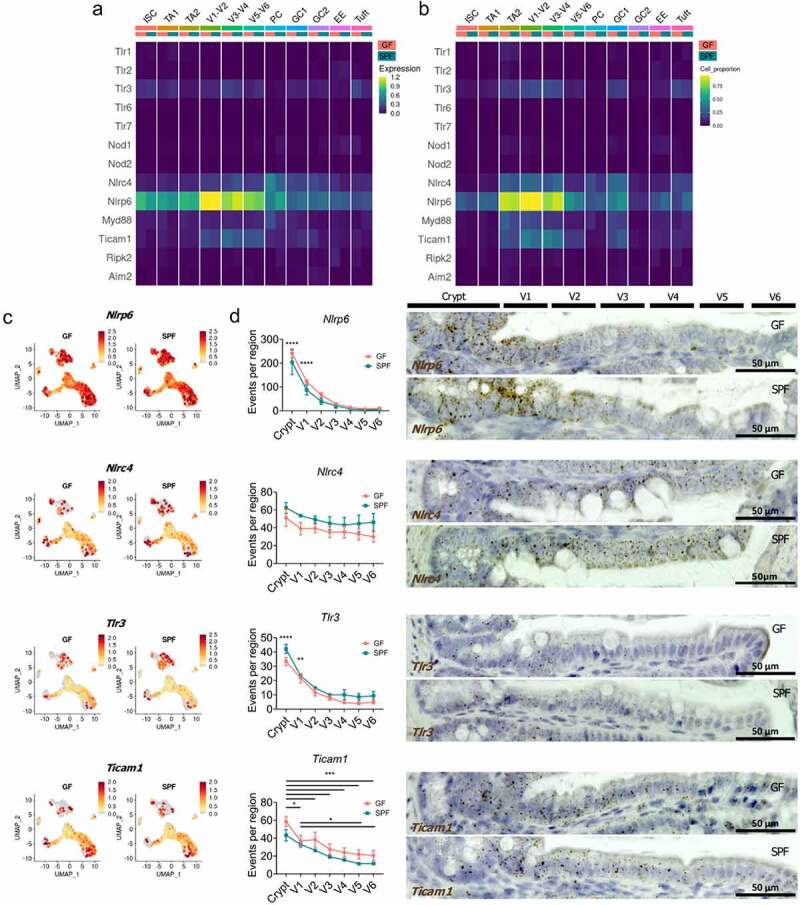
**Transcriptional mapping and validation of pattern recognition receptors (PRRs) and adaptors across intestinal epithelial cell subsets**. A) Average expression of PRRs and adaptors across epithelial cell subsets in GF and SPF. B) Proportion of cells expressing PRRs and adaptors across epithelial cell subsets in GF and SPF. C) UMAP gene expression of *Nlrp6, Nlrc4, Tlr3*, and *Ticam1* within GF and SPF epithelial cells. D) Quantification of *in situ* hybridization of *Nlrp6, Nlrc4, Tlr3*, and *Ticam1* along the crypt-villus axis. 10 jejunal/ileal crypt-villus units were assessed per mouse (n = 4–6). Stains were quantified using CellProfiler. Representative images shown. Bars represent mean ± SEM. Gene expression values are shown as normalized log_e_ expression. *, p < .05, **, p < .005, ***, p < .001, ****, p < .0001 compared to V3-V6. by two-way ANOVA, post hoc Tukey test.

### Transcriptional map of pattern recognition receptors and adaptors in the small intestinal epithelium

To determine which cell clusters have a direct capacity to sense and respond to microbes, we annotated the expression profile of pattern recognition receptors (PRRs) and their adaptors within our integrated single-cell dataset. Across all epithelial cell lineages, PRRs were expressed at low levels and were not differentially expressed with the presence of microbiota in SPF mice (Figure. 2A). *Tlr3, Nlrc4, Nlrp6* were the highest expressed PRRs along the crypt-villus axis, while *Myd88* and *Ticam1* were the highest expressed adaptors (Figure. 2A). Interestingly, *Tlr4* and *Tlr5* were not detected in any cells (Data not shown). Key crypt-villus axis distributions were observed with respect to the expression of *Tlr3, Nlrc4, Nlrp6, Myd88*, and *Ticam1*. Within the enterocyte lineages (TA2, V1-V2, V3-V4, and V5-V6), these clusters had the greatest expression and proportion of cells expressing PRRs and adaptors (Figure. 2A, B). In GCs, these genes are similarly expressed between GC1 and GC2, but expressed in a greater proportion of cells in GC1 relative to GC2 (Figure. 2A, B). This is a result of a smaller proportion of GC2 cells expressing these genes in greater abundance and suggests that there are two population of GCs differentially expressing PRRs. A similar observation was also made within the crypt compartment of ISC, PC, and TA1 cells, whereby a small proportion (<25%) of cells highly expressed these PRRs and adaptors (Figure. 2A, B).

As expression of PRRs and their adaptors was elevated in a fraction of cells within the ISC, TA1 and PC clusters, we subclustered these populations to assess their expression profiles with greater resolution. Subclustering of the ISC, TA1, and PC clusters identified a population of high mitochondria-gene expressing enterocytes, EC (Supp Figure 3A, B). Mapping the expression of all PRRs, *Tlr3* and its adaptor *Ticam1* were more associated with TA cells than ISCs (Supp Figure 3C, D). *Nlrp6* was expressed consistently across ISC, TA, and PCs (Supp Figure 3C, D) and *Nlrc4* was strongly associated with PCs (Supp Figure 3C, D). To support these data, we validated the expression distribution of *Tlr3, Ticam1, Nlrp6*, and *Nlrc4* in the jejunal/ileal crypt-villus axis. *Nlrp6* was upregulated within the crypt and V1 region (Figure. 2C, D). *Nlrc4* was expressed throughout the crypt and villus (Figure. 2C, D). *Tlr3* and *Ticam1* were upregulated within the crypt and V1 region (Figure. 2C, D). Taken together these findings suggest that progenitor cells exiting the crypt are transcriptionally primed for microbial sensing, that PRRs display regional specificity along the crypt-villus axis, and that expression of PRRs in the small intestinal epithelium is not significantly altered by microbial colonization.

**Figure 3.**
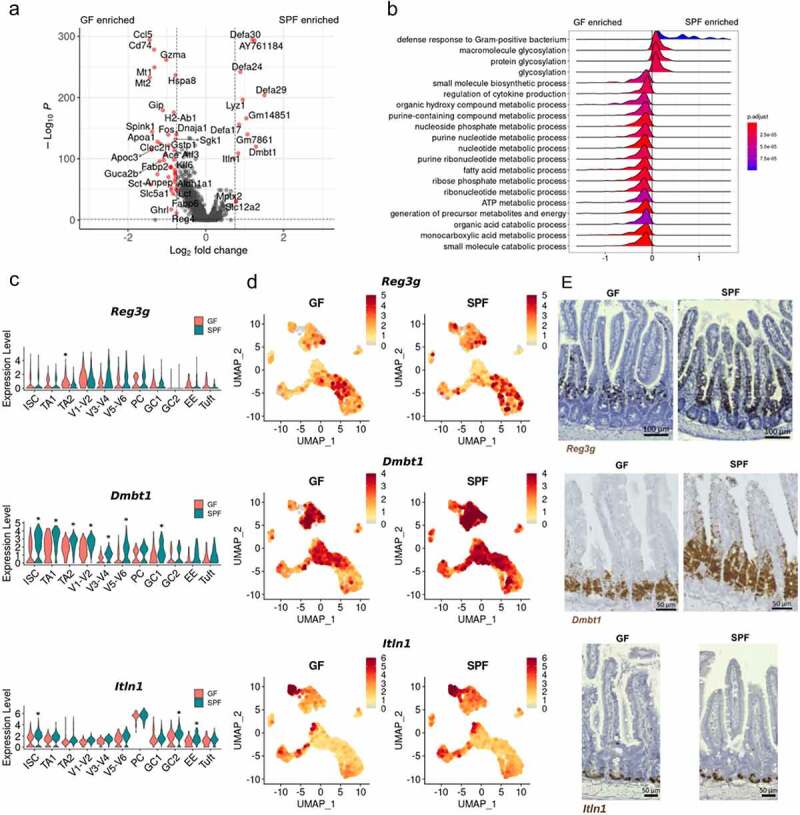
**Microbiota promotes the expression of host-defense associated genes**. A) Volcano plot highlighting differentially expressed genes between all GF and SPF epithelial cells. B) GSEA using GO: Biological Processes on ranked genes between GF and SPF epithelial cells. Gene sets are in decreasing order of their normalized enrichment score. C) Violin plots and D) UMAP visualization of *Reg3g, Dmbt1*, and *Itln1* expression across epithelial subsets in GF and SPF epithelial cells. E) In situ hybridization of *Reg3g, Dmbt1*, and *Itln1* in jejunal/ileal small intestine. Images are counterstained with hematoxylin. Representative images are shown (n = 4–6). *, p < .01 comparing GF and SPF clusters by Wilcoxon rank sum test and >0.75 Log_2_ fold change.

### The microbiota increases the expression of host defense associated genes within the small intestinal epithelium

To assess the transcriptional impact of the microbiota on the epithelium, we performed differential gene expression analysis of all GF and SPF epithelial cells. The microbiota significantly increased the expression of defensins (*Defa30, AY761184/Defa39, Defa24, Defa29*, and *Gm14851/Defa38*) and other host defense associated genes (*Lyz1, Itln1* and *Dmbt1*) (Figure 3A). Interestingly, compared to SPF, the GF epithelium upregulated the expression of a greater number of genes (Figure 3A). Using GSEA for GO biological processes in our gene list ranked by gene expression fold change, we observed that the SPF epithelium upregulated the expression of genes associated with glycosylation and the defense response to Gram-positive bacteria, while the GF epithelium had a gene expression profile associated with small molecule biosynthetic processes, regulation of cytokine production, and general metabolic processes (Figure 3B).

We further performed differential gene expression and GSEA analyses between GF and SPF cells of each cell cluster to determine cluster-specific differential gene expression profiles. Interestingly, defensins and host defense associated genes were differentially expressed across most epithelial cell lineages (Supp Figure 4). The microbiota-induced expression of genes associated with glycosylation appeared to be specifically associated with ISC and TA1 cells (Supp Figure 4). Microbiota induced genes associated with ribosomes and translation in mid/upper villus cells V3-V4 and V5-V6 (Supp Figure 4). Finally, the GF epithelium increased the expression of genes associated with cytokine production across most epithelial subsets and genes associated with responses to unfolded proteins in PCs, TA2s, and Tuft cells (Supp Figure 4).

**Figure 4.**
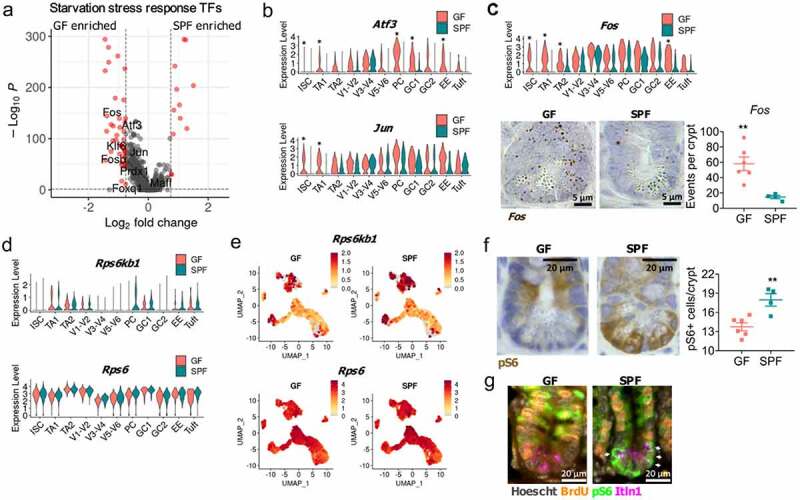
**Microbiota suppresses transcriptional nutrient stress response and activates mTOR signaling**. A) Overlay of nutrient starvation stress response transcription factor genes upon gene expression volcano plot of all GF and SPF epithelial cells. B) Violin plot of *Atf3* and *Jun* expression across GF and SPF epithelial cells. C) Violin plot of *Fos* expression across GF and SPF epithelial cells and quantification and representative image of *Fos* in situ hybridization within GF and SPF jejunal/ileal small intestinal crypts. D) Violin plots and E) UMAP visualization of *Rps6kb1* and *Rps6* epithelial subsets in GF and SPF epithelial cells. *, p < .01 comparing GF and SPF clusters by Wilcoxon rank sum test and >0.75 Log_2_ fold change. F) pS6 staining and quantification of jejunal/ileal small intestinal crypts from GF and SPF mice. G) Immunofluorescence co-staining of BrdU, pS6, and Itln1 in GF and SPF jejunal/ileal small intestinal crypts. Arrows indicate co-expression of Itln1 and pS6. Representative images shown (n = 4–6). Each point represents the mean quantification 20 crypts from one mouse (n = 4–6). Stains were quantified using CellProfiler. Bars represent mean ± SEM. **, p < .005 by two-tailed Student’s t-test.

To validate the microbially induced expression of host-defense associated genes, we performed in situ hybridization for *Reg3g, Dmbt1*, and *Itln1* in the jejunal/ileal crypt-villus axis. *Reg3g* has previously been shown to be induced in PCs by bacteria in a Myd88/Tlr-dependent manner.^31,32^ We observed an increase in *Reg3g* expression in GF TA2 cells and a trending increase in *Reg3g* expression in SPF V3-V4, V5-V6, and PCs (Figure 3C, D). These observations were validated by *in situ* hybridization, as the SPF epithelium increased expression of *Reg3g* in villus epithelial cells and within the crypts (Figure 3E). Strikingly, the microbiota dramatically increased *Reg3g* expression within the PC region (Figure 3E). *Dmbt1* expression was greatest within crypt-associated populations and was significantly increased in SPF ISC, TA1, TA2, V1-V2, V3-V4, V5-V6, and GC1 clusters compared to the GF epithelium (Figure 3C, D). In situ hybridization supported these observations as *Dmbt1* expression in SPF mice marked a greater region of TA cells and villus epithelial cells (Figure 3E). *Itln1* was most associated with PCs (Figure 3C, D) and in situ hybridization supported this observation as *Itln1* expression was associated with granulated crypt cells (Figure 3E). As defensins and *Itln1* were identified in our previous observation as differentially expressed within the epithelium, we postulate that this may be partially driven by an overall increase in PCs in the SPF epithelium as described earlier (Figure 1D).

### Microbiota-dependent metabolic signals drive pS6 signaling in the small intestinal crypts

The microbiota is known to be a critical mediator of host metabolism as it provides nutrients such as vitamin K^33^ and short chain fatty acids.^34,35^ While GF and SPF mice were fed the same diets, it is likely they are exposed to different nutrients due to the lack of microbially derived metabolites in GF mice. The overall association of the increased gene expression associated with various metabolic processes in the GF epithelium supports this hypothesis (Figure 3B). Moreover, previously identified starvation stress response transcription factors^36^ were upregulated within the GF epithelium (Figure 4A). These stress response transcription factors, such as *Atf3, Fos*, and *Jun* had increased expression within crypt cells, such as ISC and TA1 of GF mice and GF PCs had increased expression *Atf3* and trending increases in *Fos* and *Jun* (Figure 4B, C). This observation was supported by a significant increase in *Fos* staining within the jejunal/ileal crypts of GF mice (Figure 4C). As mTOR signaling is repressed by nutrient starvation stress,^37,38^ we assessed the expression of mTORC1 components, and the mTOR target, ribosomal protein *S6* and its kinase *Rps6kb1*. We observed no differences in the expression of mTORC1 components, *Mtor, Mlst8*, and *Rptor* between GF and SPF mice (Supp Figure 5). Interestingly, *Rps6kb1* trended for greater expression within SPF PC compared to GF PC, while *Rps6* was not differentially expressed within the SPF epithelium (Figure 4D, E). This suggested that the GF epithelium represses *Rps6kb1* expression and mTORC1 activity, resulting in reduced S6 phosphorylation within the crypt, primarily in PCs due to nutrient starvation stress. This was supported by a significant reduction in the number of pS6+ cells within the jejunal/ileal crypt of the GF epithelium compared to the SPF epithelium (Figure 4F) and the microbiota-dependent co-expression pS6 and Itln1, a PC marker (Figure 4G).

## Discussion

As the immediate point of contact with the microbiota, the small intestinal epithelium must integrate several environmental stimuli and respond appropriately to regulate host physiology. Characterizing this relationship at homeostasis is critical to mechanistically understand how dysbiosis and opportunistic pathogens may impact disease pathogenesis. Studying host-microbe responses with respect to specific cell-lineages has remained a challenge within the field due to difficulties in cell isolation, the rarity of specific cell types, and the lack of cell type specific antibodies. This constraint has limited our interpretation of the GF mice conventionalization experiments, the gold-standard in assessing the direct impact of specific microbiota and bacteria species. In this paper, we begin to address these limitations using scRNAseq of isolated GF and SPF small intestinal epithelium to assess the baseline microbial response in individual epithelial cell subsets.

The cellular architecture of the intestinal epithelium has been well characterized to be impacted by the microbiota in a community and species-dependent manner.^29,30,39^ By scRNAseq, we captured all epithelial cell lineages in both the GF and SPF mice, illustrating that no epithelial cell lineage is directly dependent on the presence of microbes. While we observed trends in the differences of epithelial cell subsets captured between GF and SPF samples (Supp Figure 1F), it is impossible to infer physiological differences in abundance as our crypt enrichment process may impact the proportion of villus-associated cells captured under different conditions. However, in line with previous studies, we observed a microbiota-dependent increase in PC numbers within the intestinal crypt.^29,30^ Interestingly, we did not observe differences in GC, ISC, or TA cell abundances. Trajectory inference analysis demonstrated that the differentiation pathway of ISCs to terminal ECs or secretory lineages occurs independently of microbes. With the increase in PCs and similar differentiation trajectories, our data suggest microbiota stimulate an increase in PCs from ISCs through a pathway that is intrinsic to the epithelium.

Leveraging the resolution of scRNAseq, we annotated the expression of PRRs and their adaptors within the intestinal epithelium. Interestingly, the microbiota did not impact the expression of PRRs, suggesting the intestinal epithelium is intrinsically prepared to respond to microbial ligands. In line with previous observations, PRRs and adaptors were expressed at low levels within epithelial cells.^40^ While the expression of Tlr2, Tlr4, and Tlr5 in epithelial cells has been assessed using reporter mice,^40^ we did not detect the expression of these PRRs in our dataset. This may be due to limited detection of transcripts from low-expressing genes. Notably, we observed that while Tlr3, Nlrc4, and Nlrp6 are consistently expressed within ECs, they are highly expressed in a minor proportion of GCs, ISCs, TA1 cells, and PCs.

Strikingly, differential gene expression analysis revealed GF and SPF epithelial cells have distinct transcriptional profiles. Interestingly, microbiota induced the expression of defensins, and host-defense associated genes across several epithelial cell lineages but not PCs. This may be due to low PC sampling in our dataset, preventing robust comparisons in differential gene expression. We observed a significant microbiota-driven increase of the antimicrobial peptide, *Reg3g*, as well as *Dmbt1*, a host-defense associated protein. Microbiota-dependent expression of *Reg3g* has previously been observed within PCs,^31,32^ and here we demonstrate this also occurs within mid-villus epithelial cells. Moreover, *Dmbt1*, a secreted scavenger for cysteine-rich proteins that is induced upon inflammatory stimuli and associated with Crohn’s Disease lesions^41^ was expressed in ISCs and TA cells and validated by in situ hybridization in the TA and lower villus regions. These data suggest that Dmbt1 may protect the crypt niche in a microbiota-dependent manner to prevent the entry of opportunistic bacteria. As these defense genes are expressed in a similar location to *Tlr3* and *Nlrp6*, they may be induced directly by their respective ligands. These data suggest ECs within the upper-crypt and V1 region may represent a robust source of host-defense proteins and warrants further study in the context of intestinal health and disease.

By GSEA analysis, we identified >130 genes (Supp table 1) associated with glycosylation throughout the epithelium and specifically within ISCs and TA1 cells in the homeostatic epithelium. Glycosylation of the intestinal epithelium is a critical process that provides a potent source of nutrients for the microbiota and directly maintains the symbiotic relationship.^42^ Moreover, disruption or impaired epithelial glycosylation have been associated with the development of colorectal cancer and inflammatory bowel diseases.^43–45^ While Fut1 and Fut2 have been well characterized in the murine intestine to regulate fucosylation,^46^ we identified Fut4 and Fut8 as fructosyltransferases induced by microbiota in the crypt region (Supp table 1). These data suggest that glycosylation within the crypt may impact epithelial proliferation and/or differentiation. This is supported by the observation that the modification of glycans on PCs induces greater proliferation in murine intestinal organoids.^47^ The counterintuitive expression of these direct microbiota interactors within the segregated compartment of the crypt will require further study to understand their functional role within the epithelium.

Further GSEA analysis in GF intestinal epithelium revealed several metabolism-associated gene sets. In tandem, the increased expression of starvation response transcription factors^36^ and decreased mTOR signaling by pS6 levels suggest that the absence of microbes fosters a nutritionally deficient environment for the epithelium. As such, we hypothesized that microbiota-dependent mTOR signaling was induced by the production of key microbial metabolites. Interestingly, mTOR signaling plays an important role in regulating the activity of the intestinal crypt. Chronic intestinal epithelial mTOR hyperactivation in young mice (two-month-old) induces intestinal crypt expansion and proliferation, while chronic long-term intestinal epithelial (six-month-old) mTOR hyperactivation accelerates epithelium aging and functionally decreases villus length, glucose absorption, and suppresses ISC proliferation.^48^ However, suppression of mTOR in PCs by caloric restriction has been shown to promote ISC self-renewal.^38^ In connection to these observations, our data suggest that microbiota-dependent changes in the epithelium may be driven by increased basal mTOR signaling. While we observed an increase in mTOR signaling in the SPF intestinal crypt base and PCs, we did not see a concurrent increase in ISC self-renewal as marked by significant differences in Olfm4+ ISCs or BrdU+ TA cells. This discrepancy may be attributed to the diverse mechanisms by which the microbiota and/or species-specific microbes may regulate the proliferative potential of ISCs. For instance, microbes have been shown to induce the expression of MHC class II in the intestinal epithelium^2,49^ and this may promote T helper cell cytokine interactions that directly facilitate ISC self-renewal and differentiation.^50^ Moreover, individual microbial species, such as *L. rhamnosus* and *L. reuteri* induce intestinal proliferation through NADPH oxidase 1-dependent ROS generation^51^ and dietary fructose production,^52^ respectively. While our data highlight that microbiota impact mTOR signaling in the small intestinal crypts, additional studies investigating mTOR signaling in the context of microbial metabolites will provide greater insight in leveraging microbes as drivers of epithelial restitution.

Altogether, these data highlight the cellular and transcriptional impacts of the microbiota on the small intestinal epithelium at the resolution of single cells. We present this resource that describes the transcriptional distribution of PRRs and differential transcriptional profiles of all epithelial lineages in response to microbes. We identified that microbiota induce defense-associated genes within ECs and glycosylation genes within ISCs and TA1 cells. Finally, we provide data that points to the idea that the microbiota-dependent nutrients regulate mTOR signaling within the intestinal crypt. This high-resolution comparison of GF and SPF small intestinal epithelium provides an important homeostatic comparison for future studies delineating how specific microbial communities or bacteria species impact the health of the small intestine.

## Data availability

The scRNAseq data within this study are available through the Gene Expression Omnibus GSE195742.

## Supplementary Material

Supplemental Material
